# An Exploratory Study about Factors and Outcomes Associated with the Experience of Coercive Measures in Mental Health Settings

**DOI:** 10.1007/s11126-024-10110-w

**Published:** 2025-01-16

**Authors:** Jesús Herrera-Imbroda, Vera Carbonel-Aranda, Yaiza García-Illanes, Carlos Aguilera-Serrano, Antonio Bordallo-Aragón, Edgar García-Spínola, Daniel Torres-Campos, José María Villagrán, Juan Antonio García-Sanchez, Fermín Mayoral-Cleries, José Guzmán-Parra

**Affiliations:** 1 Department of Mental Health, University General Hospital of Málaga, Biomedical Research Institute of Malaga (IBIMA), Málaga, Spain; 2 University of Málaga, Málaga, Spain; 3 Department of Mental Health, Hospital of Jerez de la Frontera, Cádiz, Spain

**Keywords:** Coercion, Mechanical restraint, Psychiatric inpatients, Patient experience

## Abstract

Coercive measures are commonly used in mental health settings despite their negative effects. The subjective experience of coercion varies widely, and its short- and long-term health impacts are not well understood. This study aimed to analyze the association between different types of coercive measures experienced during psychiatric hospitalization, the subjective experience of coercion, and both short- and long-term health outcomes. The study included 111 patients from two mental health units who experienced coercive measures (mechanical restraint, involuntary medication, or both). Perceived coercion was assessed during hospitalization. Short-term outcomes (post-traumatic stress and treatment satisfaction) were evaluated before discharge, while long-term outcomes (functionality improvement, risk of readmission, and suicidal behavior) were assessed at five-year follow-up. Perceived coercion was associated with higher post-traumatic stress (*p* < 0.001) and lower satisfaction with treatment (*p* < 0.001) in the short term. In the long term, perceived coercion showed no association with functionality, readmission risk, or suicidal behavior. However, combined coercive measures were linked to lower functionality improvement (*p* = 0.028) and higher readmission risk (*p* = 0.028) compared to involuntary medication alone. The findings suggest that efforts to reduce negative subjective experiences associated with coercive measures may improve patient satisfaction and reduce trauma risk during hospitalization. Combined coercive measures may be a risk factor for poorer long-term outcomes and should be carefully considered.

## Introduction

The use of coercive measures in mental health settings is a controversial subject and frequently involves a conflict between maintaining the user’s autonomy and the safety of those in charge of their care, their fellows, and/or the users themselves. Moreover, their use increases the risk of serious negative events that range from mental trauma and physical injuries to death. Their use could constitute a violation of patients’ human rights and patients who have experienced coercive measures frequently perceived these as antitherapeutic, punishing, humiliating, or traumatizing [1]. One source of perceived coercion in mental health settings is the use of formal coercive measures such as mechanical restraint, seclusion, and involuntary medication, among others. However, perceived coercion associated with the use of these measures and the correlates and factors associated with them has been rarely studied [2], even though are frequently experienced during psychiatric hospital admission [3].

The use of coercive measures such as mechanical restraint, seclusion, and involuntary medication has been largely associated with stress and trauma, and negative feelings in psychiatric admitted users, for example, humiliation and dehumanization [4]. Moreover, perceived coercion has been associated with less satisfaction with psychiatric treatment in more extent than the coercive measures used [5]. It has been pointed out that a dialogue between patients and therapists/staff on sensitive topics such as coercion is possible, useful, and desired by patients. Establishing transparent communication and preferences on these issues could decrease the likelihood of misunderstandings and allow for more individualized treatment options that better meet patients’ needs [6]. Likewise, perceived coercion has been associated with other negative outcomes, e.g., with increase in suicidal risk after discharge [7] and disengagement from mental health services [8]. A better understanding of the risks of perceived coercion could be important to design interventions to mitigate their possible side effects and to evaluate the real cost of the use of formal coercive measures in mental health settings.

The objective of the present exploratory study is to analyze factors associated with perceived coercion during the application of different coercive measures. The aim was to study the association between perceived coercion and satisfaction with the treatment and post-traumatic stress before discharge. Also, we wanted to study the association with other variables related with recovery after five years from the time of the admission, specifically functionality improvement, risk of readmission and suicidal behavior.

## Methods

### Procedure

During inpatient stay, previous to discharge, an assessment was conducted on users who had been subject to coercive measures (mechanical restraint, involuntary medication or both measures). The researchers were not always available, so not all eligible patients were selected. When the researchers were available (one day per week), all patients who had undergone mechanical restraint, involuntary medication or a combination of measures, and who were in condition to complete the assisted self-reporting assessment, were invited to participate. If the research and clinical staff considered that the patient was capable to be assessed, the researcher evaluated the patient’s ability to respond. The assessment was self-administered with the assistance of a researcher to solve possible doubts. Patients were recruited between May 2015 and March 2017. Follow-up data were collected solely from clinical records over a five-year period.

### Sample and Setting

The study was conducted in the Mental Health Hospitalization Units of the University Regional Hospital of Malaga (42 beds) and the General Hospital of Jerez de la Frontera (28 beds). The units are the inpatient units of the public system in the catchment area both located in general hospitals, with similar functioning and serving a population with similar characteristics. The units belong to the Andalusian Health Service, which provides universal health coverage for people living in Andalusia.

A total of 125 inpatients were asked to participate and 14 patients did not agree to participate (10.9%). Therefore, a total of 111 patients were included in the study, 93 from Malaga and 18 from Jerez de la Frontera. All assessments were conducted during the patients’ hospital admission, prior to discharge. The inclusion criteria were: (1) age between 18 and 65 years, (2) not having a diagnosis of mental retardation (F70-F79 ICD-10), (3) adequate proficiency in Spanish language to conduct the study, (4) enough capacity to understand the study and give informed consent to participate according to the nursing staff and researchers criteria, and (5) exposure to a coercive measure within 48 h prior to the assessment.

### Variables and Instruments

#### Outcome Variables

1. Before discharge, patients who met the criteria completed a self-report questionnaire assessing:

##### Satisfaction with the Treatment

*Client’s Assessment of Treatment* (CAT) [9]: the scale evaluates a patient’s satisfaction with their hospital treatment. The users rate each item on a scale of 0 to 10. Due to the lack of a validated Spanish version, the scale was translated by the authors for this study. The seven items are straightforward in linguistic structure, focusing on aspects such as perceived appropriateness of treatment, therapist engagement, respect, and overall helpfulness. The scale have and adequate internal consistency [10] and predictive [11] and factorial [12] validity.

##### Event Related Stress

*Davidson Trauma Scale* (DTS) [13]: this questionnaire has 17 items with 5 categories from “Never” to “Daily”, to evaluate each of the 17 symptoms collected in the DSM-IV on Post-Traumatic Stress Disorder (PTSD) in subjects who have suffered a stressful event. Each item has two evaluation scales, one for frequency and one for intensity. The scale is validated in Spanish by Bobes et al. [14]. The instrument was applied considering the coercive measures experienced during the psychiatric hospitalization. This instrument has shown adequate psychometric properties for screening when the traumatic event is close to the evaluation [15].

2) Five-year follow-up assessment:

Three variables were collected from clinical records. (1) Change in functionality was calculated subtracting the punctuation in the Global Assessment of Functioning (GAF) after five-years from assessment to the GAF punctuation at discharge. (2) Inpatient readmission during follow-up, and (3) suicidal behaviour (suicidal attempt or suicidal ideation recorded in the medical history) during the follow-up.

### Independent Variable and Covariables

#### Perceived Coercion

*Short Version of the Coercion Experience Scale* (CES-18) [16, 17]: This instrument has 18 items with 5 categories from “Nothing” to “Extremely”. The instrument has adequate internal consistency, factorial, convergent and divergent validity [17].

##### Covariables

Socio-demographic variables (gender, age, study centre and nationality) and clinical diagnosis according to ICD-10 were also analysed. Type of coercive measure applied was also considered:

*Mechanical Restraint*: It is defined as the application of fastening devices to limit physical mobility to prevent damage to the patient, other people, and/or the physical environment that surrounds them. 

*Involuntary Medication*: Defined as any medication administered (oral or injectable) against the patient’s will. In our study, while most administrations were injectable, in cases where oral medication was used, patients took it under pressure or an implied threat of consequences if they did not comply, which they perceived as a coercive measure. 

*Combination of measures*: Both measures described above applied at the same time.

### Statistical Analysis

For the bivariate analysis, logistic regression was used to compare the group between high and low perceived coercion regarding sociodemographic and clinical variables. We fitted multivariate linear regression models in which the dependent variables were satisfaction with the treatment and perceived trauma (CAT and DTS). To analyze the assumptions of the models, we used a scatter plot to verify the linear association of the variables, the Breusch-Pagan test to check heteroskedasticity, Shapiro-Wilks test to verify the normality of the residuals and variance inflation factor to check collinearity. When functionality change at the five years follow-up was used as dependent variable the homoscedasticity assumption was not met, so we used a robust regression model (*robustbase* package and the *lmrob* function in R). When we used as dependent variable the psychiatric readmission and the presence of suicidal behavior in the follow-up, we carried out multivariate logistic regression. To assess the goodness of fit of the model the Hosmer and Lemeshow test was used. In all the multivariate models were introduced as confounder the following variables: gender, age, nationality, the study center and the type of coercive measure used. We set a level of confidence of 95%. The analyses were carried out with software R version 4.1.3.

## Results

Most of the participants were male (68.5%), and the mean age of the sample was 37.81 years. A 59.5% had a diagnosis of a psychotic disorder (ICD-10). Regarding differences in the clinical and sociodemographic variables among the groups with high and low perceived coercion, there were statistically significant differences regarding the coercive measure applied (*p* < 0.001), with low risk of perceived coercion when involuntary medication was applied in comparison with combined measures (OR = 0.169, *p* < 0.001). More information about the sample is shown in Table 1.

**Table 1:** Characteristics of the study sample and comparisons between groups with high and low perceived coercion

Variables	Total *n* = 111	High perceived Coercion *n* = 55	Low perceived coercion *n* = 56	*p*
Age (Mean, SD)	37.81 (12.14)	36.78(11.47)	38.82 (12.79)	OR = 0.986, *p* = 0.375
Gender (N, %)				
Male Female	76 (68.5) 35 (31.5)	37 (48.7) 18 (51.4)	39 (51.3) 17 (48.6)	OR = 0.896, *p* = 0.788
Center (N, %) Malaga Jerez	93 (83.8) 18 (16.2)	44 (47.3) 11 (61.1)	49 (52.7) 7 (38.9)	OR = 0.571, *p* = 0.288
Educational level (N, %) Primary or less Secondary or university	72 (64.9) 39 (35.1)	34 (47.2) 21 (53.8)	38 (52.8) 18 (32.1)	OR = 0.767, *p* = 0.506
Employment status (N, %) Unemployment Other situation	58 (52.3) 53 (47.7)	27 (46.6) 28 (52.8)	31 (53.4) 25 (47.2)	OR = 0.768, *p* = 0.509
Nationality (N, %) Spanish Others	98 (88.3) 13 (11.7)	46 (46.9) 52 (53.1)	9 (69.2) 4 (30.8)	OR = 0.393, *p* = 0.141
Type of coercive measure Involuntary medication Mechanical restraint Combined measure	41 (36.9) 32 (28.8) 38 (34.2)	11 (26.8) 18 (56.3) 26 (68.4)	30 (73.2) 14 (43.8) 12 (31.6)	*p* < 0.001 OR = 0.169, *p* < 0.001 OR = 0.593, *p* < 0.295
Diagnosis according ICD-10 (N, %) Substance disorders (F10-F19) Psychotic disorders (F20-F29) Affective disorders (F30-F39) Personality disorders (F60-F69) Others	9 (8.1) 66 (59.5) 25 (22.5) 5 (4.5) 6 (5.4)	4 (44.4) 34 (51.5) 13 (52.0) 2 (40.0) 2 (33.3)	5 (55.6) 32 (48.5) 12 (48.0) 3 (60.0) 4 (66.7)	*p* = 0.903 OR = 1.600, *p* = 0.668 OR = 2.125, *p* = 0.402 OR = 2.167, *p* = 0.418 OR = 1.333, *p* = 0.819

The regression linear models showed a negative significant association between perceived coercion experienced and satisfaction with the treatment (B=-0.332, *p* < 0.001) and a positive significant association with posttraumatic stress (B = 0.077, *p* < 0.001, *Root Square of Davidson Trauma Scale as dependent variable) during the admission. The association maintain the significance after controlling for possible cofounders (See Table 2).

**Table 2:** Association between perceived coercion and satisfaction with the treatment and trauma at discharge

**Model 1 (Dependent variable = Satisfaction with the hospitalization treatment, CAT)** ^1^					
**Variables**	**Coeficients**	**Error**	**t**	***p***	**VIF** ^*^
Intercept	59.659	5.980	9.977	< 0.001	
Perception of coercion (CEV)	-0.302	0.072	-4.176	< 0.001	1.175
Coercive measure					1.238
Combined measure	Ref				
Involuntary medication	2.226	2.808	0.793	0.430	
Mechanical restraint	2.641	2.817	0.937	0.351	
Gender					1.080
Male	Ref				
Female	1.778	2.411	0.738	0.462	
Centre					1.160
Malaga	Ref				
Jerez	-3.490	3.150	-1.108	0.271	
Nacionality					1.090
Spanish	Ref				
Other	-0.501	3.500	-0.143	0.887	
Age	0.116	0.095	1.223	0.224	1.133
**Model 2 (Dependent variable = Root Square of Davidson Trauma Scale)** ^2^					
**Variables**	**Coeficients**	**Error**	**t**	***p***	**VIF** ^*^
Intercep	-0.349	0.960	-0.363	0.717	
Perception of coercion (CEV)	0.074	0.012	6.361	< 0.001	1.175
Coercive measure					1.238
Combined measure	Ref				
Involuntary medication	-0.367	0.451	-0.815	0.417	
Mechanical restraint	0.229	0.452	0.507	0.613	
Gender					1.080
Male	Ref				
Female	0.187	0.387	0.482	0.631	
Centre					1.160
Malaga	Ref				
Jerez	-0.015	0.506	-0.029	0.977	
Nacionality					1.090
Spanish	Ref				
Other	0.074	0.562	0.131	0.896	
Age	0.004	0.015	0.282	0.778	1.133

^1^R^2^=0.233. Breusch Pagan Homoscedasticity test = 0.165. Shapiro Wilks test of residuals = 0.395

^2^R^2^=0.350. Breusch Pagan Homoscedasticity test = 0.409. Shapiro Wilks test of residuals = 0.901

Comparing clinical outcomes in the five years follow-up there were not a significant association between perceived coercion during the admission and change in functionality (B = 0.030, *p* = 0.745), risk of readmission (OR = 1.015, *p* = 0.206) and suicidal behaviour (OR = 0.983, *p* = 0.235) at follow-up. This lack of significant association remains in the multivariate models (see Table 3). However, there was an association between the type of coercive measure applied and change in functionality (involuntary medication vs. combined measure; B = 7.805, *p* = 0.029) and marginally significant with risk of readmission (involuntary medication vs. combined measure; OR = 0.404, *p* = 0.067) in the five-year follow-up. The association was significant with the two dependent variables in the multivariate models (see Table 3).

**Table 3:** Association between perceived coercion and functionality improvement, psychiatric admission and suicidal behavior during 5 year follow up

**Model 1 (Dependent variable = Follow-up Change in Functionality, GAF)** ^1^				
**Variables**	**Coeficients**	**Error**	**t**	***p***
Intercept	-10.696	7.755	-1.379	0.171
Perception of coercion (CEV)	0.129	0.101	1.277	0.205
Coercive measure				
Combined measure	Ref			
Involuntary medication	9.250	4.142	2.234	0.028
Mechanical restraint	1.622	4.405	0.368	0.714
Gender				
Male	Ref			
Female	-0.885	4.370	-0.203	0.840
Centre				
Malaga	Ref			
Jerez	-4.244	4.377	-0.970	0.334
Nacionality				
Spanish	Ref			
Other	-0.835	6.993	-0.120	0.905
Age	0.011	0.162	0.066	0.948
**Model 2 (Dependent variable = Inpatient Admission)** ^2^				
**Variables**	**OR**	**Error**	**Wald**	***p***
Intercep	0.219	1.430	1.128	0.288
Perception of coercion (CEV)	1.021	0.015	2.002	0.157
Coercive measure				0.055
Combined measure	Ref			
Involuntary medication	0.250	0.586	5.585	0.018
Mechanical restraint	0.374	0.574	2.939	0.086
Gender				
Male	Ref			
Female	1.311	0.473	0.329	0.567
Centre				
Malaga	Ref			
Jerez	6.740	0.672	8.072	0.004
Nacionality				
Spanish	Ref			
Other	6.129	0.749	5.867	0.015
Age	0.947	0.020	7.564	0.006
**Model 3 (Dependent variable = Follow-up Suicidal Behaviour)** ^3^				
**Variables**	**OR**	**Error**	**Wald**	***p***
Intercept	0.012	1.848	5.794	0.016
Perception of coercion (CEV)	0.989	0.016	0.488	0.485
Coercive measure				
Combined measure				
Involuntary medication	1.778	0.690	0.697	0.404
Mechanical restraint	2.126	0.667	1.280	0.228
Gender				
Male				
Female	3.018	0.638	3.001	0.083
Centre				
Malaga				
Jerez	1.301	0.866	0.092	0.761
Nacionality				
Spanish				
Other	2.033	1.113	0.406	0.524
Age	1.037	0.021	2.930	0.087

^1^ Robust linear regression

^2^ Hosmer and Lemeshow test: X^2^ = 8.312, *p* = 0.404

^3^ Hosmer and Lemeshow test: X^2^ = 5.231, *p* = 0.733

## Discussion

The results of the study show that a higher perception of coercion during the application of coercive measures during the hospital admission was a risk factor for presenting less satisfaction with hospital treatment and for developing post-traumatic stress symptoms. There was no significant relationship between the perception of coercion and the change in functionality, the risk of readmission and the risk of suicidal behavior at five years. However, presenting combined coercive measures was associated with a higher risk of readmission and a lower increase in functionality during the five-year follow-up of the study.

These results regarding satisfaction with the treatment are consistent with those of two recent systematic reviews, based mainly on qualitative studies, in which the perception of coercion was related to the perception of the role of staff and the perception of treatment received [4, 18]. Thus, it is not surprising that a higher perception of coercion with respect to the application of coercive measures is associated with lower satisfaction with the treatment. Also, an Italian longitudinal study found that the perception of coercion at admission was related to less satisfaction with treatment [19]. Another study associated the perception of coercive measures with the users’ view of psychiatric treatment [20]. Likewise, a relationship has been found between the perception of coercion at admission and the therapeutic relationship [21]. Increase the satisfaction with the psychiatric treatment is gaining more importance over time and can influence the therapeutic relationship and effectiveness of treatment in the long term [5]. Multiple studies have found that proper treatment of staff, information provided, respect, and subsequent debriefing [22–24] influences the perception of coercion, which may explain the relationship with treatment´s satisfaction and indicates that efforts to improve staff procedures can decrease perceived coercion and increase satisfaction with treatment.

It was also found that a higher perception of coercion was associated with a higher risk of experiencing post-traumatic stress, which is consistent with a study that found that perceived institutional restraint was associated with psychological distress [25] and with the validation study of the Coercive Experience Scale in which there was a high correlation between the perception of coercion and post-traumatic stress disorder screening scale score [16]. A recent study found that in a quarter of patients the use of mechanical restraint was related to the presence of post-traumatic stress [26]. In our study, the perception of coercion had more influence on the presence of post-traumatic stress than the type of measure used.

Regarding the results at follow-up, the perception of coercion was not related to changes in functionality, and the risk of readmission or suicide. Other studies have found that the perception of coercion has been related to the subjective perception of recovery [27] but in our study it was not related to general indicators of recovery. Other studies have found that the use of increased restraint has not been associated with changes in functionality or short-term clinical improvement [28]. However, the use of combined measures compared with involuntary medication was associated with a less pronounced improvement in function and a higher risk of readmission. Therefore, the use of combined measures was a risk factor for a worse prognosis during the disorder. One possible explanation is that combined use of coercive measures could be due to a more severe form of the disorder and therefore explain the worse prognosis. Other studies have also shown that the use of mechanical restraint and seclusion is associated with risk of readmission [29, 30].

### Limitations

This was an observational study and it is not possible to establish a causal relationship between independent and outcome variables. The application of coercive measures is different between health services in different countries and even in different Autonomous Communities in Spain. Moreover, perceived coercion could vary between different cultures. For that, generalizations of the results could be problematic. This study utilized a convenience sample due to the limited availability of researchers in the units; therefore, a prior sample size calculation was not conducted. The available sample size may limit the statistical power of the study to detect small effects, which should be taken into account when interpreting the results. Additionally, the study did not consider certain confounding variables, such as diagnostic categories, which could influence both the application of coercive measures and patients’ subjective perceptions. Finally, the study did not assess many organizational factors that may have influenced the subjective experiences of the coercive measures.

## Conclusions

The study shows that the perception of coercion during the application of coercive measures such as involuntary medication, mechanical restraint, or combined measures, was related to lower satisfaction with the hospitalization treatment and to a higher risk of post-traumatic stress. These results indicate that organizational and humanizing measures during the application of coercive measures are recommended to improve patient satisfaction and reduce the risk of trauma. Likewise, the results indicate that the use of combined coercive measures should be avoided during hospitalization, as they may be a risk factor for poorer recovery and a worse long-term prognosis.

### Research Implications

The results suggest that decrease the perceived coercion during hospitalization could improve satisfaction with treatment and prevent trauma. This study highlights the importance of protect human rights and dignity of users of mental health hospitalization units and to include, as much as possible, the user in the decision-making process related with their treatment. Moreover, the study shows the importance to avoid the use of coercive measures and coercion in general in psychiatric settings. Likewise, the results suggest that combined coercive measures should be avoided as its use is associated with higher risk of readmission and worse functionality in the long term. If alternative interventions to prevent the use of coercive measures have failed, it is recommended to implement further interventions to prevent patients from suffering negative experiences and increase perceived coercion.

## Data Availability

The dataset could be available under reasonable request to the corresponding author.
